# Synthesis and Evaluation of Technetium-99m-Labeled pH (Low) Insertion Peptide Variant 7 for Early Diagnosis of MDA-MB-231 Triple-Negative Breast Cancer by Targeting the Tumor Microenvironment

**DOI:** 10.3389/fonc.2022.869260

**Published:** 2022-04-21

**Authors:** Yuehua Chen, Yuan Su, Xufeng Pang, Xiaoxia Song, Wanjun Zhao, Mingming Yu

**Affiliations:** Department of Nuclear Medicine, The Affiliated Hospital of Qingdao University, Qingdao, China

**Keywords:** triple-negative breast cancer, early diagnosis, tumor microenvironment, pH (low) insertion peptides (pHLIP), molecular imaging, Technetium-99m, small-animal SPECT/CT

## Abstract

**Objective:**

To prepare technetium-99m (^99m^Tc)-labeled pH (low) insertion peptide variant 7 [pHLIP (Var7)] and carry out small-animal single-photon-emission computed tomography (SPECT)/computed tomography (CT) imaging of tumor-bearing nude mice *in vivo* to study its value in the early diagnosis of triple-negative breast cancer (TNBC).

**Methods:**

The pHLIP (Var7) sequence was synthesized *via* solid-phase peptide synthesis. Four amino acids, Gly-(D)-Ala-Gly-Gly, were attached to the N-terminus of pHLIP (Var7) to form a strong chelating group containing an N4 structure. The peptide was labeled with ^99m^Tc using a direct labeling method. We determined the *in vitro* binding fraction of ^99m^Tc-pHLIP (Var7) to MDA-MB-231 cells. Serial biodistribution studies and small-animal SPECT/CT imaging in MDA-MB-231 TNBC-bearing mice were performed using ^99m^Tc-pHLIP (Var7).

**Results:**

The radiochemical yield and purity of ^99m^Tc-pHLIP (Var7) were 99.49 ± 0.17% and 99.63 ± 0.44%, respectively. The radiochemical purity was still more than 96% after 24 h in serum. The binding fraction of ^99m^Tc-pHLIP (Var7) to MDA-MB-231 cells continuously increased in an acidic environment and was significantly higher than the cell-binding fraction (P < 0.01) at pH = 7.4 and the cell-binding fraction (P < 0.01) of ^99m^Tc-kVar7 at different pH values (pH = 6.0, 6.5, 7.0 and 7.4) at each time point (P < 0.01). The distribution of ^99m^Tc-pHLIP (Var7) in tumors at each time point was significantly greater than that of ^99m^Tc-kVar7 (P < 0.01). SPECT/CT imaging was largely consistent with the biodistribution results; the tumor was clearly imaged at each time point after injection of ^99m^Tc-pHLIP (Var7) but could not be imaged after injection of ^99m^Tc-kVar7.

**Conclusion:**

^99m^Tc-pHLIP (Var7) showed a high radiochemical yield and stability and was highly concentrated in tumor tissues. Although there was strong radioactive background in the abdomen of tumor-bearing nude mice, it did not hinder early diagnosis of TNBC.

## Introduction

The tumor microenvironment (TME) of almost all solid tumors is acidic, with pH values as low as 6.0 (1, 2). The acidic TME is stable and not affected by tumor clonal selection, making it a promising tumor detection target (2). The pH (low) insertion peptide (pHLIP) family can target the acidic TME, spontaneously form an α-helix structure, and insert it into tumor cell membranes in an acidic extracellular environment (3). In previous studies (4, 5) with iodine-125-labeled pH (low) insertion peptide variant 7 [PHLIP (Var7)], single-photon-emission computed tomography (SPECT)/computed tomography (CT) imaging of MDA-MB-231 triple-negative breast cancer (TNBC) mice confirmed that pHLIP (Var7) can target MDA-MB-231 TNBC. However, the iodine-labeled compound is easily deiodinated, and its half-life is unsuitable for imaging. Technetium-99m (^99m^Tc), which is better for SPECT imaging, was applied for labeling and imaging in the present study. In addition, a novel ^99m^Tc labeling method was used: four amino acids, Gly-(D)-Ala-Gly-Gly, that formed an N4 structure were used as a chelating group and linked to the N-terminus of pHLIP (Var7) during peptide synthesis to realize direct ^99m^Tc labeling. This method simplifies the labeling process and raises the radiochemical yield and purity (6).

## Materials and Methods

### Materials

#### Main Instruments

The main instruments employed in this study were a Chirascan plus ACD spectropolarimeter (Applied Photophysics, UK), CRC-55tR radiopharmaceutical dose calibrator (Capintec Inc., USA), Wizard 1480 gamma counter (PerkinElmer Instruments Inc., USA), Scan-RAM Radio-HPLC/TLC scanner (LabLogic, UK), and U-SPECT +/CT small-animal SPECT/CT imaging system (MILabs, The Netherlands).

#### Main Reagents

The main reagents applied in this study were ^99m^Tc-pertechnetate (Shanghai Xinke Pharmaceutical Co., Ltd., China), stannous chloride (Sinopharm Chemical Reagent Co., Ltd., China), isoflurane (Shenzhen RWD Life Technology Co., Ltd., China), and hydrochloric acid (Sinopharm Chemical Reagent Co., Ltd., China).

### Synthesis and Characterization of Peptides

pHLIP (Var7) and the control sequence kVar7 (7) were synthesized by Shanghai Science Peptide Biological Technology Co., Ltd. (Shanghai, China) using solid-phase peptide synthesis. To facilitate ^99m^Tc labeling, four amino acids, Gly-(D)-Ala-Gly-Gly, were attached to the N-terminus of pHLIP (Var7) and kVar7 to form a strong chelating group containing an N4 structure. To prevent any spatial obstruction, gamma-aminobutyric acid (GABA) was introduced as a spacer between the N4 structure and pHLIP (Var7)/kVar7. The modified pHLIP (Var7) and kVar7 sequences were as follows:pHLIP (Var7), GaGG-GABA-ACEEQNPWARYLEWLFPTETLLLEL-NH_2_; kVar7, GaGG-GABA-ACEEQNPWARYLKWLFPTKTLLLKL-NH_2_.

pHLIP (Var7) and kVar7 were purified *via* high-performance liquid chromatography (HPLC) using a Shimadzu-LC2010 instrument (Shimadzu Corporation, Japan) equipped with a Kromasil 100-5C18 chromatographic column (AkzoNobel, Sweden) (5 µm, 4.6 × 150 mm). The peptides were eluted with a gradient from 25 to 85% solvent A (0.1% trifluoroacetic in 100% acetonitrile) and 75 to 15% solvent B (0.1% trifluoroacetic in 100% water) at 1.0 ml/minute, with monitoring at 220 nm. The identities of pHLIP (Var7) and kVar7 were confirmed by mass spectrometric analysis using an Agilent 6125B mass spectrometer (Agilent Technologies, Inc., USA).

### Circular Dichroism (CD)

Next, 7 µmol/L PHLIP (Var7) and 2 mmol/L 1-palmitoyl-2-oleoyl-*sn*-glycero-3-phosphocholine (POPC; diameter ≤ 50 nm) were dissolved in 10 mmol/L phosphate buffer (PB) at pH = 8 and equilibrated at room temperature for at least 10 h. The pH value of the solution was adjusted to 4.0 with 0.1 mol/L HCl.

CD spectra of pHLIP (Var7) were recorded at pH = 8.0 and pH = 4.0 in a 1-mm-path-length quartz cuvette using a spectropolarimeter. The experimental settings were as follows: bandwidth, 1 nm; wavelength range, 190–260 nm; wavelength step size, 1 nm; time-per-point, 0.5 s; temperature, 25°C.

### Preparation of ^99m^Tc-pHLIP (Var7) and ^99m^Tc-kVar7

A total of 1 mg peptide powder was dissolved in 1 mL 0.02 M phosphate-buffered saline (PBS). Ten microliters of the above-prepared solution was added to 90 μL PBS. Then, 20 μL of 1 mg/mL stannous chloride (in 0.01 M HCl), 20 μL sodium phosphate solution (8.2 mg/mL), and 74 MBq fresh ^99m^Tc-pertechnetate were added. The mixture was then placed in a 90°C water bath for 15 min. The product was purified in a Sephadex G25 column (1.0 × 25 cm) and eluted with PBS (0.1 M, pH = 7.4). The radiochemical yield and specific activity of ^99m^Tc-pHLIP (Var7) and ^99m^Tc-kVar7 were calculated.

The radiochemical purity of ^99m^Tc-pHLIP (Var7) and ^99m^Tc-kVar7 was determined by thin-layer chromatography (TLC). A thin polyamide layer was used as the stationary phase, and acetonitrile was used as the developing solvent. A sufficient amount of the labeled product was aspirated through a capillary pipette and spotted at the origin of the thin polyamide layer. The thin polyamide layer was air-dried and vertically placed in a test tube with developing solvent. The saturated thin polyamide layer was removed, dried, and placed on the TLC scanner for testing.

### *In Vitro* Stability of ^99m^Tc-pHLIP (Var7) and ^99m^Tc-kVar7

^99m^Tc-pHLIP (Var7) and ^99m^Tc-kVar7 were placed in PBS at room temperature (25°C) and in fresh human serum at 37°C. Samples were taken to test the radiochemical purity at 1, 4, 6, and 24 h after labeling.

### Cell Culture and Xenograft Model

MDA-MB-231 human breast cancer cells (Chinese Academy of Sciences) were cultured in an incubator at 37°C with 5% CO_2_ in high-glucose Dulbecco’s modified Eagle’s medium containing 10% fetal bovine serum and 1% antibodies (penicillin–streptomycin mixture).

Animal experiments were approved by the ethics committee of Qingdao University, and the management of animals followed ethical standards. We built an MDA-MB-231 tumor model using four- to- five-week-old female BALB/c nude mice (Shanghai SLAC Laboratory Animal Co., Ltd, China). To do so, 0.1 mL of a cell suspension (50 µL PBS + 50 µL Matrigel) containing 1 × 10^6^ MDA-MB-231 cells was subcutaneously inoculated into the right axilla of each mouse.

### Binding of ^99m^Tc-pHLIP (Var7) and ^99m^Tc-kVar7 to MDA-MB-231 Cells

a. When MDA-MB-231 cells grew to more than 90% confluence, they were digested. The cell concentration was adjusted to approximately 1.0 × 10^5^ cells/mL, and 1 mL of the cell solution was added to each well of a 12-well plate and cultured overnight in a 37°C cell incubator.

b. The medium was discarded, and the cells were washed with PBS twice. Then, 2 mL of fresh medium and 37 kBq ^99m^Tc-pHLIP (Var7) or ^99m^Tc-kVar7 (20-30 μL) were added to each well. We adjusted the pH values of the wells to four levels (pH 7.4, 7.0, 6.5, and 6.0).

c. After culturing for 30, 60, 90, 120, and 180 min, the supernatant was aspirated into a supernatant tube, and each well was washed two times with PBS at the corresponding pH (7.4, 7.0, 6.5, or 6.0). The cleaning solution was also added into the supernatant tube. The cells were digested fully with 0.25% trypsin, and the cell suspension was aspirated into a cell tube and washed twice with PBS. The cleaning solution was also added to the cell tube. A γ counter was applied to measure the radioactivity count of the cell tube (B) and supernatant tube (F). The cell-binding fraction of ^99m^Tc-pHLIP (Var7) or ^99m^Tc-kVar7 = B/(B+F) × 100%.

### *In Vivo* Biodistribution

When tumor diameters reached 0.8–1.0 cm, 24 tumor-bearing nude mice were selected for an *in vivo* biodistribution experiment. Each nude mouse was injected with approximately 1480 kBq/200 μL ^99m^Tc-pHLIP (Var7) or ^99m^Tc-kVar7 through the tail vein. The mice were sacrificed humanely 1 h, 2 h, 4 h, and 6 h after injection, and their tissues and organs, such as the brain, heart, lung, liver, kidney, stomach, small intestine, blood, muscle, bone, and tumor, were weighed and measured for their radioactivity count. The results are expressed as the percentage of injected dose per gram of tissue (%ID/g).

### Small-Animal SPECT/CT Imaging

When tumor diameters reached 0.8–1.0 cm, six tumor-bearing nude mice were selected for imaging. Each nude mouse was injected with approximately 11.1 MBq/200 μL ^99m^Tc-pHLIP (Var7) or ^99m^Tc-kVar7 through the tail vein. The tumor-bearing mice were anesthetized with 2% isoflurane at 1 h, 2 h, 4 h, and 6 h after injection and were fixed on the imaging bed of a small-animal SPECT/CT imaging system in the prone position. A pinhole collimator was applied. The main parameters were a matrix size of 256 × 256, magnification power of 1.45, and acquisition time at each time point of 10 min.

### Statistical Analysis

SPSS 24.0.0.0 (IBM) statistical software was applied for data processing. Quantitative data are expressed as the mean (M) ± standard deviation (SD). Variables were compared *via* one-way analysis of variance. P < 0.05 was deemed significant.

## Results

### Peptide Synthesis

pHLIP (Var7) and kVar7 were successfully synthesized *via* solid-phase peptide synthesis. HPLC analysis of pHLIP (Var7) showed the formation of several compounds consisting of a major product (98.12%, retention time of 12.85 min) accompanied by two smaller peaks at retention times of 12.75 and 13.24 min. HPLC analysis of kVar7 showed the formation of several compounds consisting of a major product (98.03%, retention time of 11.82 min) accompanied by two smaller peaks at retention times of 11.47 and 12.33 min.

MS analysis of pHLIP (Var7) showed two mass peaks with m/z values of 1131.70 [(M+3H)3H+] and 1697.13 [(M+2H)2H+]. MS analysis of kVar7 showed two mass peaks with m/z values of 1130.80 [(M+3H)3H+] and 1695.70 [(M+2H)2H+]. The measured molecular weights of pHLIP (Var7) and kVar7 (3392.10 and 3389.40, respectively) were consistent with the theoretical molecular weights (3391.80 and 3388.98, respectively).

### CD Spectroscopy

**Figure 1:**
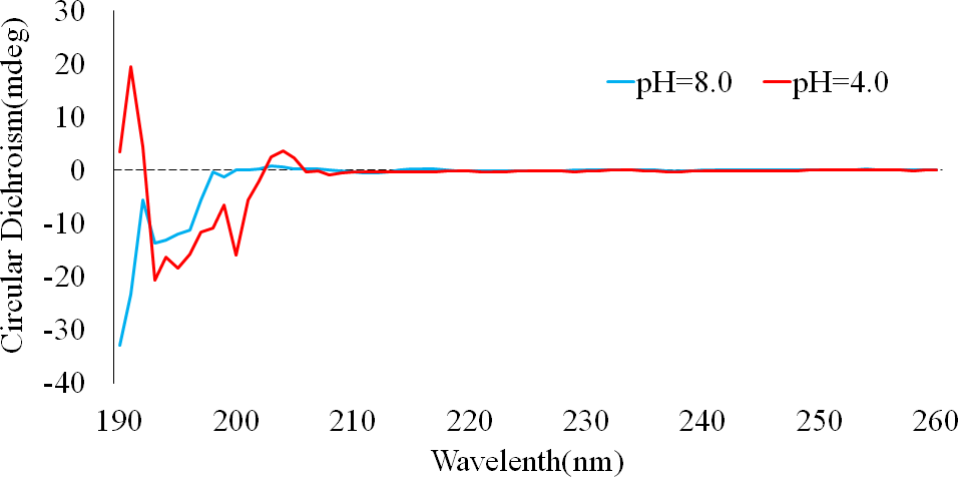
Circular dichroism of pHLIP (var7) at pH 4.0 (red line) and pH 8.0 (blue line).

The secondary structure of pHLIP (Var7) was determined by CD at pH 8.0 and 4.0. **Figure 1** shows that pHLIP (Var7) exhibited a typical pH-dependent transition from a nonstructural conformation to an α-helical structure when the pH decreased from 8.0 (blue line) to 4.0 (red line) in the presence of POPC.

### Preparation of ^99m^Tc-pHLIP (Var7) and ^99m^Tc-kVar7

^99m^Tc-pHLIP (Var7) had a radiochemical yield of 99.49 ± 0.17% and purity of 99.63 ± 0.44%. ^99m^Tc-kVar7 had a radiochemical yield of 99.46 ± 0.14% and purity of 99.63 ± 0.35%. The specific activities of ^99m^Tc-pHLIP (Var7) and ^99m^Tc-kVar7 were both 7.36 ± 0.01 MBq/μg.

### *In Vitro* Stability of ^99m^Tc-pHLIP (Var7) and ^99m^Tc-kVar7

**Figure 2:**
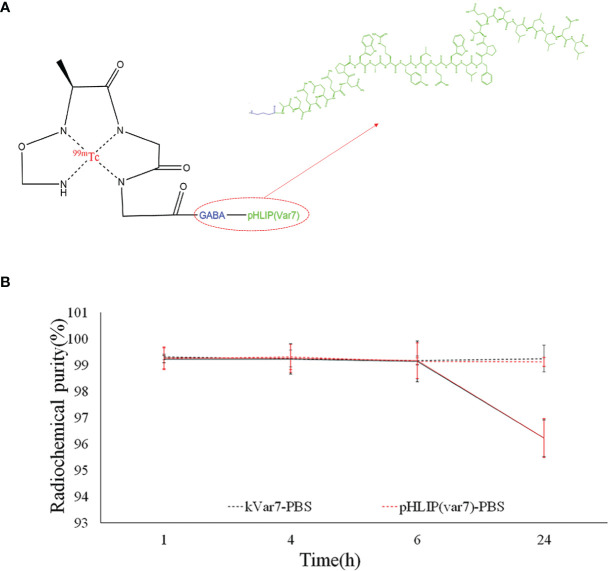
**(A)** N4 structure of ^99m^Tc-pHLIP (Var7). **(B)** Stability of ^99m^Tc-pHLIP (Var7) and ^99m^Tc-kVar7 in PBS at room temperature and in fresh human serum at 37°C.

The radiochemical purity of both ^99m^Tc-pHLIP (Var7) and ^99m^Tc-kVar7 was > 99% in PBS at room temperature at 24 h and > 96% in fresh human serum at 37°C at 24 h (**Figure 2**).

### Binding Fractions of ^99m^Tc-pHLIP (Var7) and ^99m^Tc-kVar7 to MDA-MB-231 Cells

**Figure 3:**
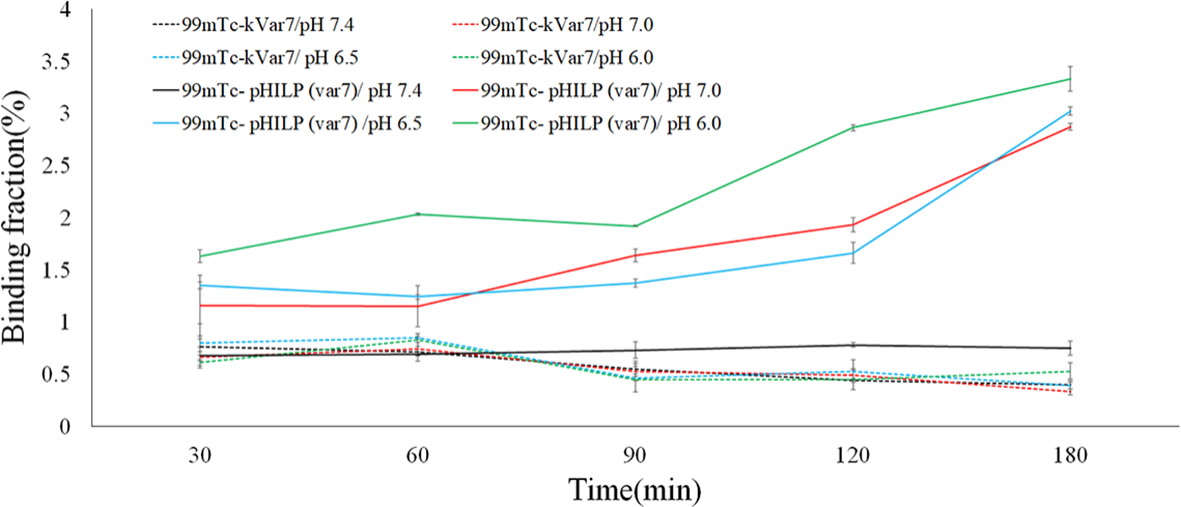
Binding fraction of ^99m^Tc-pHLIP (Var7) and ^99m^Tc-kVar7 to MDA-MB-231 cells at different times and at different pH values (n=3).

At pH = 6.0, 6.5, and 7.0, the binding fractions of ^99m^Tc-pHLIP (Var7) to MDA-MB-231 cells steadily increased and reached the highest levels at 180 min, which were 3.33 ± 0.12%, 3.02 ± 0.04%, and 2.87 ± 0.03%, respectively. At pH = 7.4, the binding fractions of ^99m^Tc-pHLIP (Var7) to MDA-MB-231 cells remained low at each time point (30, 60, 90, 120, and 180 min). The binding fractions of ^99m^Tc-kVar7 to MDA-MB-231 cells also remained low at each time point (30, 60, 90, 120, and 180 min) at all tested pH values (6.0, 6.5, 7.0, and 7.4; **Figure 3**).

The cell-binding fractions of ^99m^Tc-pHLIP (Var7) at pH = 6.0, 6.5, and 7.0 at various time points (30, 60, 90, 120, 180 min) were significantly higher than the cell-binding fractions at pH = 7.4 (P < 0.01) and than the cell-binding fractions of ^99m^Tc-kVar7 at pH = 6.0, 6.5, 7.0, and 7.4 (P < 0.01).

### Biodistribution

**Figure 4:**
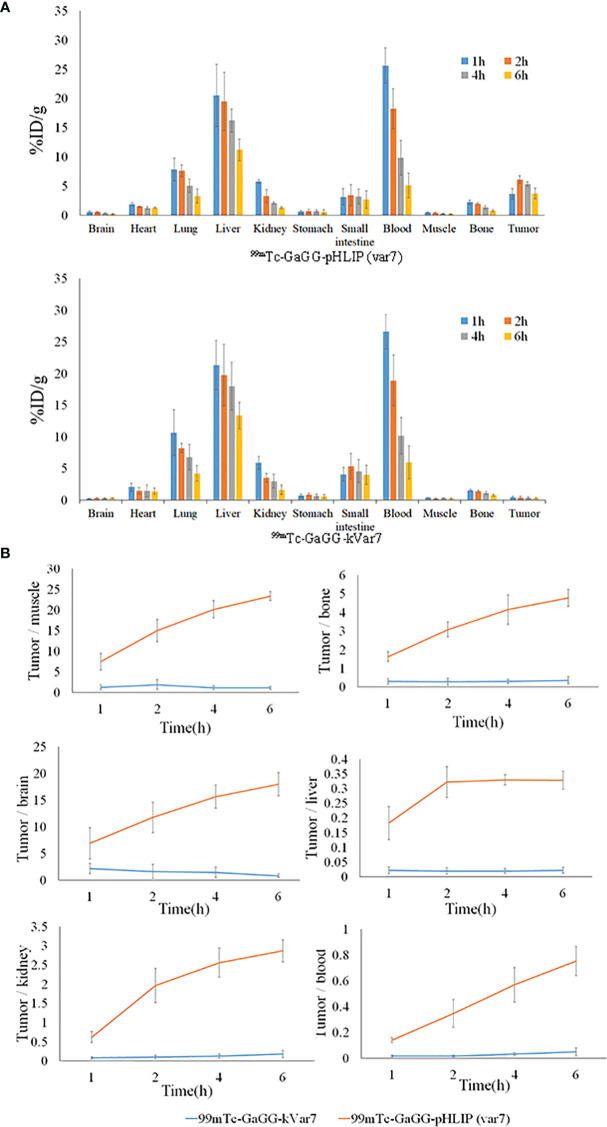
The biodistribution **(A)** and T/NT ratio **(B)** of ^99m^Tc-pHLIP(Var7) and ^99m^Tc-kVar7 in mice with MDA-MB-231 tumors (n=3).

The distribution of ^99m^Tc-pHLIP (Var7) in tumors at 1, 2, 4, and 6 h was 3.63 ± 0.92, 6.11 ± 0.66, 5.33 ± 0.40, and 3.72 ± 0.94% ID/g, respectively, all of which were higher than the distribution levels of ^99m^Tc-kVar7 in tumors (P < 0.01). Of the measured off-target organs, ^99m^Tc-pHLIP (Var7) and ^99m^Tc-kVar7 showed high distribution levels in the liver and blood (**Figure 4A**; n = 3).

The tumor/nontumor (T/NT) ratio increased over time in the ^99m^Tc-pHLIP (Var7) group, while no significant increasing trend was seen in the T/NT ratio in the ^99m^Tc-kVar7 group. The T/NT ratio in the ^99m^Tc-pHLIP (Var7) group was higher at each time point than in the ^99m^Tc-kVar7 group (P < 0.05; **Figure 4B**; n = 3).

### Micro-SPECT/CT Imaging

**Figure 5:**
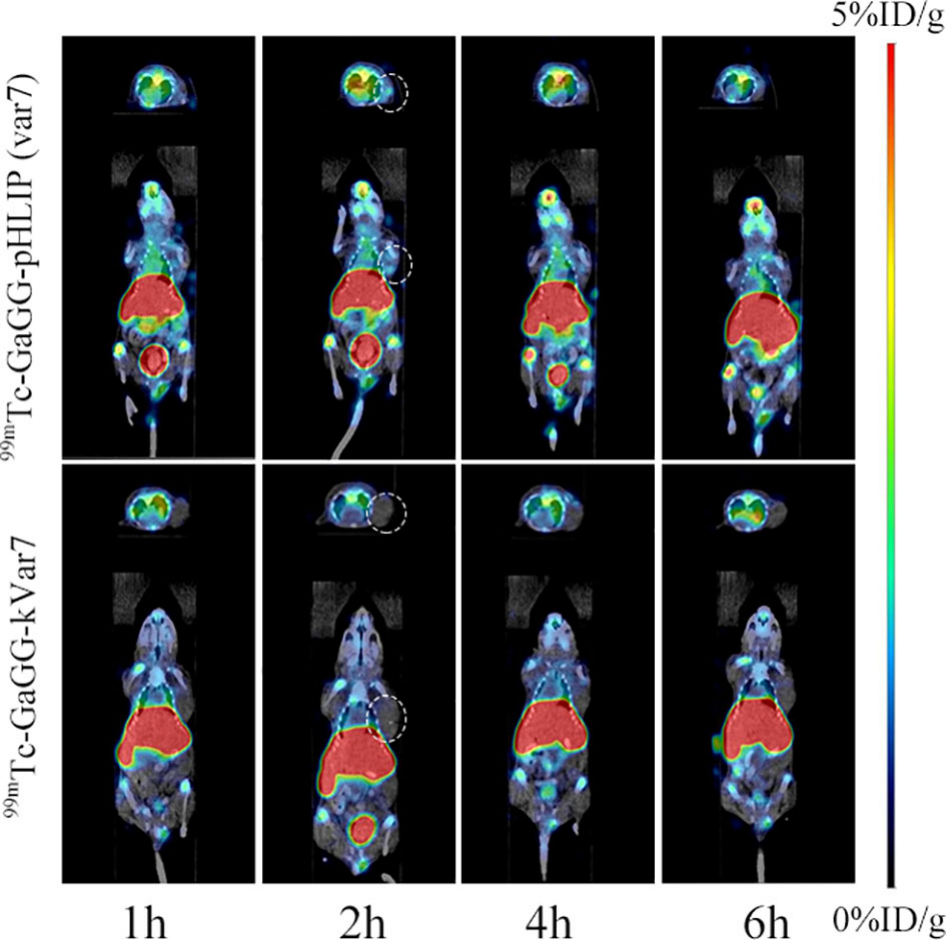
SPECT/CT imaging of ^99m^Tc-pHLIP (Var7) and ^99m^Tc-kVar7 in the MDA-MB-231 tumor model (n=3).

SPECT/CT imaging was performed at 1, 2, 4, and 6 h after the injection of ^99m^Tc-pHLIP (Var7) or ^99m^Tc-kVar7 (**Figure 5**; n = 3). Tumors were clearly imaged at each time point after injection of ^99m^Tc-pHLIP (Var7) but not after injection of ^99m^Tc-kVar7. The liver was clearly imaged in both groups at each time point.

## Discussion

TNBC can easily metastasize and has a poor prognosis. Early diagnosis of TNBC can effectively improve the prognosis. In daily clinical practice, the imaging diagnosis of breast cancer is typically performed *via* mammography, magnetic resonance imaging, and ultrasound (8). Since TNBC is more likely to occur in young women (9), the relatively dense breast tissue can interfere with the detection of tumor lesions using conventional imaging methods. In this study, ^99m^Tc—which has a low cost, suitable half-life, and radiation energy for imaging—was used for the first time to label pHLIP (Var7), which can target the TNBC TME (4, 5, 10, 11), for SPECT imaging. SPECT imaging is characterized by high sensitivity and low cost; thus, it is widely used. Combined with ^99m^Tc-pHLIP (Var7), which exhibits a typical pH-dependent transition from an unstructured conformation to an α-helix structure in an acidic environment (confirmed by CD spectroscopy), SPECT could be used for functional imaging of TNBC and is expected to be used for early diagnosis and screening of TNBC.

At present, for ^99m^Tc labeling, indirect labeling is primarily used, which is relatively complex and leads to an insufficient radiochemical yield and poor stability *in vitro*. In this study, by referring to the labeling technology described by Zhao et al. (6), four amino acids, Gly-(D)-Ala-Gly-Gly, were attached to one end of the peptide as a chelating group for direct ^99m^Tc labeling. The labeling method was simple and easy. The radiochemical yield and purity of the radioactive molecular probe in this study were both greater than 99%, indicating that the labeling method can achieve a high radiochemical yield and purity, and the radioactive probe can be used for *in vivo* imaging without purification. The 24-h radiochemical purity of the molecular probe in fresh human serum at 37°C *in vitro* was more than 96%, suggesting that the radioactive probe has high resistance to proteases in the blood and high stability. The application of small peptides as bifunctional coupling agents has the following advantages: they can be directly synthesized *via* solid-phase peptide synthesis, and they can form stable compounds with ^99m^Tc (12).

^99m^Tc-pHLIP (Var7) showed a high binding fraction to MDA-MB-231 cells in an acidic environment, significantly higher than the binding fraction of the control peptide ^99m^Tc-kVar7, suggesting that the N-terminal labeling of pHLIP (Var7) with ^99m^Tc did not change the ability of pHLIP (Var7) to insert itself into tumor cell membranes in acidic environments. The insertion of pHLIP family peptides into the lipid bilayer of cell membranes is triggered by low pH values. In acidic environments, the negatively charged amino acid residues (Asp or Glu) in pHLIP are protonated, and an α-helix forms for insertion into the cell membrane (3, 13). kVR7, a pH-insensitive peptide obtained by replacing the protonated Glu residue in the insertion region of pHLIP (Var7) with a positively charged Lys residue, does not target acidic tissues (7). Therefore, kVR7 was used as the control peptide in this study.

Biodistribution assays showed that ^99m^Tc-pHLIP (Var7) and ^99m^Tc-kVar7 had slow blood clearance, suggesting that they might bind strongly to plasma proteins and/or red blood cells. Complexes formed by binding are not conducive to probe elimination and lower the image quality. In future studies, we will consider changing the characteristics of probe molecules (such as molecular weight, amino acid sequence, and charge number) to reduce their binding to plasma proteins and/or red blood cells. The high distribution of the probes in the kidney suggests that the probes might be excreted through the urinary system. One study found (14) that the pHLIP family is mainly excreted through the urinary system because the kidneys are the main excretion route for low-molecular-weight proteins, and normal urine is weakly acidic, facilitating excretion of the pHLIP family. That study (14) showed that the addition of a weakly alkaline bicarbonate buffer to the drinking water of mice reduced the accumulation of pHLIP in their urinary system. Urine may be appropriately acidified to improve the ability of the probe to be excreted in the urine and thus reduce the radiological background in the image.

The distribution of both probes in the liver was high. The liver uptake of fluorescence-labeled pHLIP (Var7) (15) was much lower than that of ^99m^Tc-pHLIP (Var7), likely because ^99m^Tc-labeled pHLIP (Var7) bound strongly to intrahepatic tissue proteins and ^99m^Tc labeling changed the original elimination characteristics of pHLIP (Var7), thus, it was partially excreted in bile. Biodistribution data further showed that although there was a very strong radioactive background in the abdomen of mice, the distribution of ^99m^Tc-pHLIP (Var7) in tumor tissue was significantly higher than that in muscle, bone, and other tissues. Based on the biodistribution characteristics of the probes, it could be predicted that primary TNBC would be easily identifiable in SPECT images, which would be valuable for the early diagnosis of TNBC.

The results of SPECT/CT imaging of tumor-bearing nude mice were largely consistent with the *in vivo* biodistribution trend. The tumors in mice were imaged clearly at various time points after injection of ^99m^Tc-pHLIP (Var7), while the tumors were not imaged after injection of ^99m^Tc-kVar7, suggesting that ^99m^Tc-pHLIP (Var7) was electrically neutral in the acidic TME due to protonation of the negatively charged Glu residue, and the peptide changed from a disordered structure to an α-helix structure and inserted itself into tumor cell membranes. ^99m^Tc-kVar7 remained positively charged in the acidic TME and did not convert to an α-helix to insert itself into tumor cell membranes. Although the intense radioactive concentration in the livers of the tumor-bearing nude mice interfered with the detection of abdominal metastases, it did not hinder the imaging of primary TNBC lesions enriched in ^99m^Tc-pHLIP (Var7); thus, ^99m^Tc-pHLIP (Var7) can potentially be used for early diagnosis of TNBC and breast cancer screening in high-risk groups.

## Conclusion

^99m^Tc-pHLIP (Var7) has a high radiochemical yield and high stability and shows high accumulation in TNBC. Although ^99m^Tc-pHLIP (Var7) has a strong radioactive concentration in the abdomen, this does not prevent the imaging of primary TNBC. ^99m^Tc-pHLIP (Var7) is expected to become a valuable radioactive molecular probe for early TNBC diagnosis and screening.

## Data Availability Statement

The datasets presented in this study can be found in online repositories. The names of the repository/repositories and accession number(s) can be found in the article/**Supplementary Material**.

## Ethics Statement

The animal study was reviewed and approved by ethics committee of Qingdao University.

## Author Contributions

YC, YS, XP, XS, and WZ performed the experiments and wrote the paper. MY designed and supervised the research. All authors read and approved the final manuscript.

## Funding

This study was funded by the Natural Science Foundation of Shandong Province (Grant No. ZR2021MH038), Shandong Provincial Medical and Health Science and Technology Development Program (Grant No. 202009040347) and the Affiliated Hospital of Qingdao University “Clinical Medicine+X” Scientific Research Project (Grant No. QDFY+X202101013).

## Conflict of Interest

The authors declare that the research was conducted in the absence of any commercial or financial relationships that could be construed as a potential conflict of interest.

## Publisher’s Note

All claims expressed in this article are solely those of the authors and do not necessarily represent those of their affiliated organizations, or those of the publisher, the editors and the reviewers. Any product that may be evaluated in this article, or claim that may be made by its manufacturer, is not guaranteed or endorsed by the publisher.
