# Supplementary material for: Synthesis and Evaluation of Technetium-99m-Labeled pH (Low) Insertion Peptide Variant 7 for Early Diagnosis of MDA-MB-231 Triple-Negative Breast Cancer by Targeting the Tumor Microenvironment
Source: Front Oncol. 2022 Apr 21;12:869260. doi: 10.3389/fonc.2022.869260 (PMC9069674; doi:10.3389/fonc.2022.869260)
Supplement: Supplementary file 1 [file DataSheet_1.pdf]

Supplementary Material

A

B

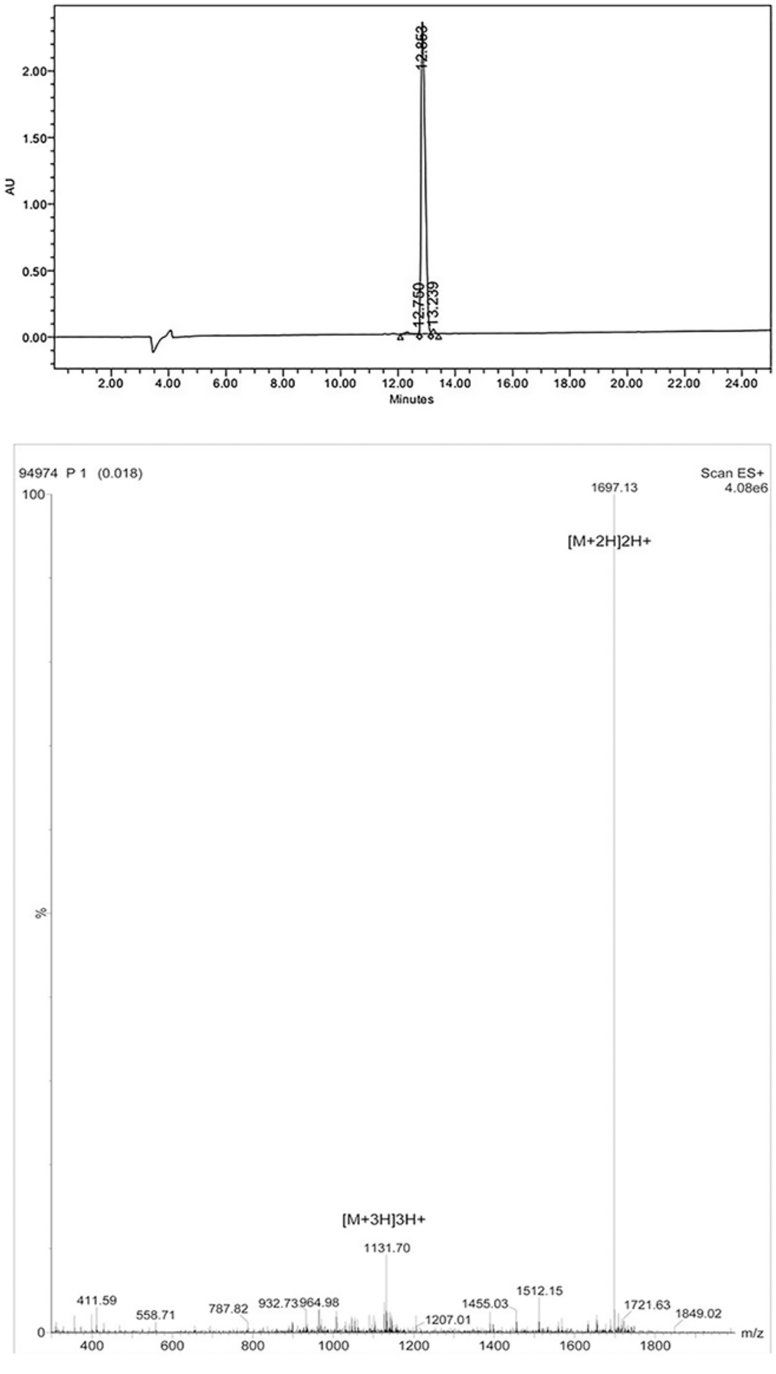

Supplementary Figure 1. The HPLC(A) and MS spectra(B) of pHLIP (Var7).

A

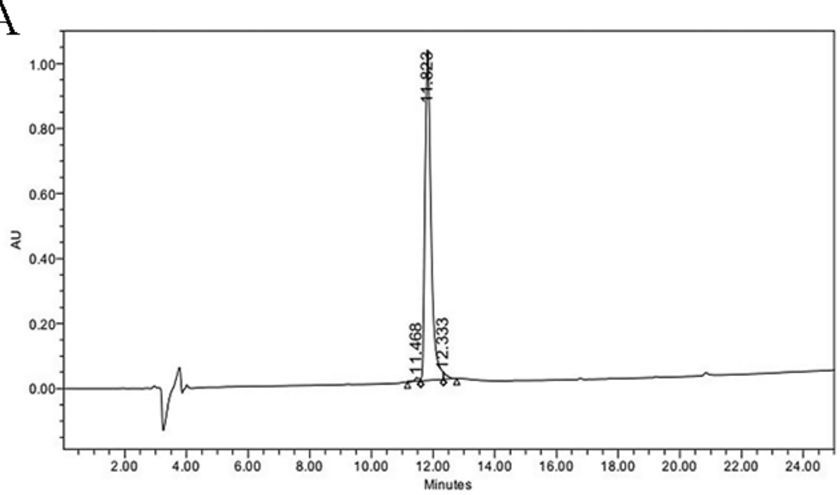

B

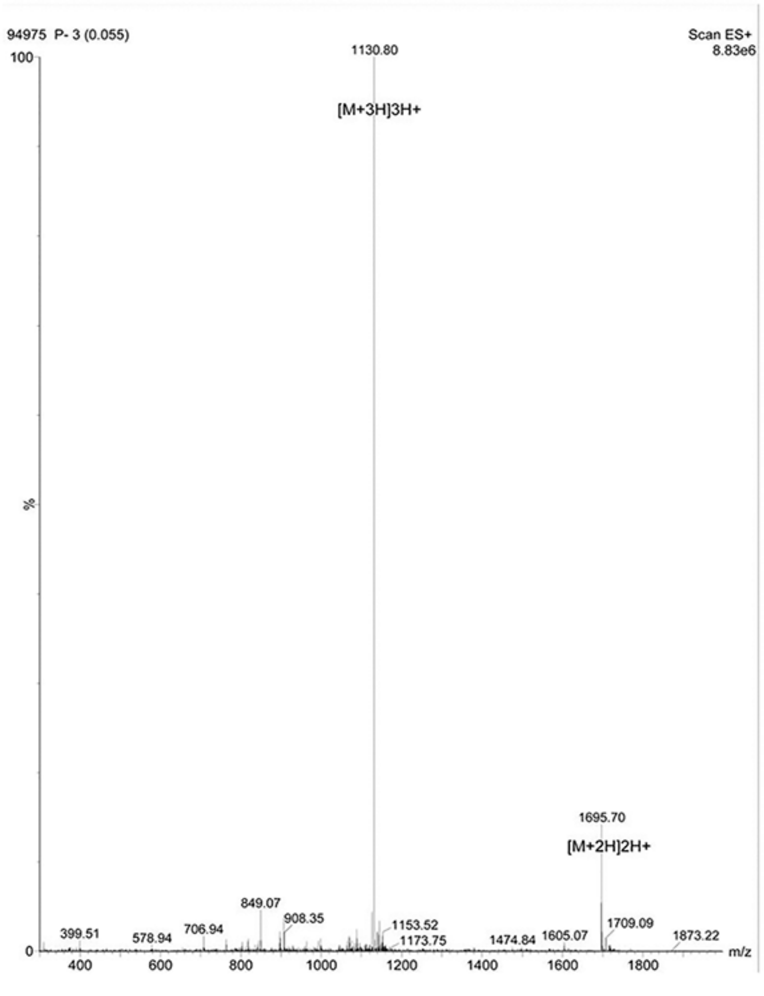

Supplementary Figure 2. The HPLC(A) and MS spectra(B) of kVar7.
